# Artificial intelligence is an oxymoron: The importance of an organic body when facing unknown situations as they unfold in the present moment

**DOI:** 10.1007/s00146-021-01311-z

**Published:** 2021-11-05

**Authors:** Jakob Svensson

**Affiliations:** School of Arts & Communication, Malmö University, Malmö, Sweden

**Keywords:** Artificial intelligence, Body, Cusa, Nonconsciousness, Organic, Presence

## Abstract

Departing from popular imaginations around artificial intelligence (AI), this article engages in the I in the AI acronym but from perspectives outside of mathematics, computer science and machine learning. When intelligence is attended to here, it most often refers to narrow calculating tasks. This connotation to calculation provides AI an image of scientificity and objectivity, particularly attractive in societies with a pervasive desire for numbers. However, as is increasingly apparent today, when employed in more general areas of our messy socio-cultural realities, AI- powered automated systems often fail or have unintended consequences. This article will contribute to this critique of AI by attending to Nicholas of Cusa and his treatment of intelligence. According to him, intelligence is equally dependent on an ability to handle the unknown as it unfolds in the present moment. This suggests that intelligence is *organic* which ties Cusa to more contemporary discussions in tech philosophy, neurology, evolutionary biology, and cognitive sciences in which it is argued that intelligence is dependent on having—and acting through—an organic body. Understanding intelligence as organic thus suggests an oxymoronic relationship to artificial.

## Introduction

I am finding myself at a showcase at the tech, innovation, film and music festival South by Southwest (SXSW) in Austin Texas. It is March 2019, and I am listening to a legendary singer from the Fugees, Wyclef Jean (who was also a presidential candidate in Haiti in 2010). The showcase is titled as his latest mixtape “Wyclef goes back to School”. This is based on a project in which he discovers and showcases young new talents. But before we actually get to listen to these talents, Jean himself gets to speak: *There is nothing that can beat the human in the present, no data no AI no nothing!*. Then he all of the sudden jumps down of the stage and puts his face very close to a woman in the first row of the enthusiastic audience. Naturally, she starts to flush and also becomes frightened of his sudden move. *Now that is what I am talking about*, exclaims Jean now facing the audience, *that reaction would not happen if I would have sent her my face pic on drop box.* The audience laughs. Then he goes on about he discovered the RnB group Destiny’s Child by meeting them face to face in a hotel room: *No AI, no nothing,* he repeats, and continues, *to be inspired, we need to be present*.

I start with this episode from SXSW19 because it is telling of our time that a public pop-cultural icon like Wyclef Jean decides to start his presentation on music with references to AI (artificial intelligence). This episode exemplifies that discussions around AI are taking place outside of science, technology, engineering and mathematics (the so-called STEM disciplines) and thus not only concern those involved in the latest advances in machine learning and artificial neural networks. Apart from a technological innovation, AI is also a *rhetorical construct* (Gunkel 2020, p. 15), or a *discursive practice* (Agree 1997, p. 140) and popular cultural have given rise to imaginations around AI for quite some time. Penny (2013, p. 149) refers to imaginations of AI as *techno-mythology*, informed by SciFi (Science Fiction) fantasies and disseminated via popular media. The movie industry has produced a wide variety of feature films in which the topic of AI has been addressed. The genre of SciFi has arguably had a huge influence, not only on the imaginations of the general public, but also on the imaginations of those engineering AI (Svensson 2021). As Chun (2006) has convincingly argued, cyberspace existed in the public’s imagination before it became a regular practice. Furthermore, popular imaginations play a central role for shaping the meaning of technologies. They reveal the socio-cultural issues tied up with technological innovations (Hayles 1999, p. 22). Imaginaries are indeed productive and cannot be dismissed as false believes (Bucher 2017, p. 31). As Gunkel (2020, p. 17) puts it, words matter, and AI is as much a product of how we talk about it, as it is a product of programming and engineering. Hence, we need to also include SciFi writers, filmmakers and public intellectuals when discussing AI, their promises and limitations.

Tegmark (2017, p. 78) suggests that the entrance of AI into the mindset of larger segments of the population was due to explosive improvements in computer memory and computer power. Together with an increase of collected and stored data (Cukier and Mayer-Schoenberger 2013) it is today possible to expose powerful computers for massive amounts of data to “learn” (i.e., to find patterns and correlate). AI is accompanied with a promise of a quick and relatively painless fix to problems the globe is currently facing (such as corona virus-tracing, climate change and increased polarization). While such *technological solutionism* can be contested, the contemporary progress in areas such as protein prototyping, automated image- and voice recognition are no doubt both impressive and imagination provoking.

However, AI does not only evoke rosy images of a better and brighter tomorrow. Within the popular realm we also find public intellectuals warning us of a super-intelligent AI running amok (Bostrom 2014) or being used in the service of undemocratic forces (see prelude in Tegmark 2017). Then we have the whole singularity scare forwarded by science superstar Stephen Hawkins. Scandals such as Cambridge Analytica, browsing millions of Facebook profiles and using their data traces for political purposes in elections, have increased concerns around surveillance and data protection. Many have heard about China’s Social Credit system of mass surveillance and control using facial recognition and big data analysis technology for among other things decisions on banking credits, insurance premiums and possibilities for travelling abroad. There are increasing concerns of using systems in for example predictive policing, university ranking, job application, credit, and insurance worthiness, as also Critical Data scholars have highlighted (see for example O’Neill 2016).

Returning to SXSW and Wyclef Jean, I believe he is on to something when he talks about the body in the present moment and connects that to the limits of AI. Can AI deal satisfactorily with the messiness of the present given that the data AI calculations are based upon, are most often (if not always) biased, and most often (if not always) based on traces from the past (for an outline of how I conceive of data, see Svensson and Poveda 2020)? Can AI then be fully intelligent? In this article, I will dwell on this question by attending to the *I* in the *AI* acronym.

The 

## AI and intelligence: a short history

The term AI was coined at the now-famous Dartmouth conference during the summer 1956 by mathematician and computer scientist McCarthy (Engster and More 2020).^1^ Since then AI has endured at least two *winters*, when investments dried up. Today it could be argued we are in the height of an AI summer. Thanks to machine learning there is a boom in interest and investment around AI (Bostrom 2014, p. 9; Gunkel 2020, p. 11), also thanks to developments in computer power, memory and big data (as mentioned in Section 1).

The premise of AI is the construction of computers that exhibit intelligence (Agree 1997, p. 139). As intelligence was/is thought to be located in the brain (conceived of as separable from the rest of the body, so-called *computationalist cognitivism*, see Penny 2013, p. 151), the quest to create an artificial brain was what motivated AI pioneers (Engster and More 2020). The invention of the digital programmable computer in the 40s coincided with ideas of human intelligence as mechanical manipulation of symbols. Hence, scientists began to seriously discuss the possibility of building an electronic brain. Already Wiener (1947, p. 14), in his famous *Cybernetics* theory, talked about the computing machine as a model of the brain with computer switches being equivalent of brain neurons. Ensmenger (2012, pp. 28, 33), in his history of computer software, refers to how computers in numerous publications in the late 40s were described as *giant* or *mechanical brains*. More than 60 years later, in 2011, the now-famous *Google Brain* project begun, and in Europe the EU funds the *Human Brain Project*. The area of neural networks, with related fields of deep and machine learning, is also inspired by biological neurons in the brain. Given this continued preoccupation with replicating a brain, perhaps it would be more correct to label the field AB (Artificial Brain) instead of AI?

The use of the very term intelligence—which is thought of as being located inside of the brain—was important for the field of AI to kick off. At Dartmouth, when the decision to name the field was taken, the use of intelligence was particularly seductive. If they would have called it *symbolic processing* or *analytical computing* (as were two other candidates), it would arguably not have had the same impact (Engster and Moore 2020). But this reduces intelligence to manipulation of symbols rather than enaction in the human lifeworld (Hayles 1999, preface xi). Still, the reference to intelligence and its appeals to SciFi was pivotal to secure funding. As Weizenbaum has underlined, the simplistic notion of intelligence that has dominated, both scientific and popular thought, is in part responsible for AI’s “perverse grand fantasy to grow" (Weizenbaum 1976, p. 203).

Here it is important to mention Turing and his famous Turing test (sometimes also called the Imitation Game). For Turing (1950), a machine is intelligent when it can *pass* as a human.^2^ His Turing test played a role in the movie *Ex Machina* from 2014 (directed by Garland) which is set around to get a humanoid robot, *Ava,* to pass the test and thus be considered intelligent. Ava succeeds even in getting a human to fall in love with her, a theme that was already explored in Jonze’s 2013 acclaimed movie *Her* in which a man falls in love with his AI-powered voice assistant.

Turing’s take on intelligence has been influential. Early AI scientists referred to technologies that were intended to *simulate* human capabilities, i.e., pass as humans, when explaining their research. Today public intellectuals such as Tegmark (2017, p. 52) defines Artificial General Intelligence (AGI) as the ability to accomplish any goals *at least as well as humans*. Bostrom (2014, p. 23) similarly defines Human-Level Machine Intelligence (HLMI) as carrying out human professions *at least as well as a typical human*. But as Searle showed in his famous *Chinese Room* thought experiment (explained in Gunkel 2020, p. 40) simulation (or passing) is not the same as understanding and thus provides a narrow view of intelligence. Furthermore, using the human as a referent for intelligence, AI scholars paradoxically forwards a very anthropocentric understanding of intelligence.

You might wonder whether we humans want to be fooled by AIs as it seems we actively attribute human-like characteristics to machines. Weizenbaum showed already in the 60s with his famous chatbot *Eliza* (one of the first to attempt the Turing Test) that participants were quick to form tight relationships with Eliza. While intended to reveal the superficiality of communication between man and machine, Weizenbaum was appalled by how far humans are willing to go to allow machines to pass as humans (see interview by Wendt 2015, pp. 90–91). Having nothing to say of her own (as she merely picks up on keywords in the questions asked to her), Eliza was still convincing enough to pass as a human (Gunkel 2020, p. 36).

Apart from viewing machines as separate entities that may (or may not) pass as humans, another popular imagination revolves around human–machine symbiosis such as in the famous movie *RoboCop* from 1987. This theme has been popularized through the cyborg figure and famous intellectuals such as Haraway (1991, p. 149) advocating for less monstrous connotations to animal-machine fusions. Today, historian Harari’s (2015) account of an emerging *Homo Deus* is among the more acclaimed. He suggests that the body might merge with the computer with the consequence that our species, *Homo sapiens*, will evolve into a new and more powerful *Homo deus* (or *posthuman* as Hayles 1999, labels it when computation becomes the foundation of being). This is about envisioning humans as information processing machines, our bodies as a very advanced algorithm. However, in our organic bodies it seems we cannot realize our full potential. Therefore, Harari predicts that either we will merge with a non-organic system or that our whole brain with its neural networks will be replaced by intelligent software. This is about rolling technological, mathematical, informational and biological processes into one, crumbling “the veil between the organic and the manufactured” (Harari 2015, p. 200).^3^ And if flesh is data incarnate, “why not go back to the source and leave the perils of physicality behind” as Hayles (1999, p. 37) rhetorically asks. The contrast between the body’s limitations and cyberspace’s power is also highlighted in cyberpunk classic *Neuromancer* (Gibson 1984) to which I will return to later in the article.

Instead of becoming upgraded with computers, or machines passing as humans, others have warned us of an intelligence explosion, the so-called *singularity*, referring to the moment in time when mahines become conscious, break out from their pre-designed goals and start controlling their own destiny. Mathematics professor Vinge, who coined the term, was actually also a SciFi author. Already Moravec (1988) in his well-known *Mind Children* argued that humans were on the brink to be replaced by intelligent machines. Bostrom (2014) has written about this in terms of *superintelligence* in his best-selling book with the same name. Superintelligence according to him is something we need to fear and make sure we can control. He defines superintelligence as any intelligence that greatly exceeds the cognitive performances of humans in virtually all domains of interest (something Weiner was onto already in 1948, p. 7). An example of this we find in the *Prometheus* tale that opens Tegmarks (2017) best-selling book *Life 3.0*. This is a story of the rise of a superintelligence that first starts to earn money, publishes films, generates a new tech boom, manipulates electoral politics and ultimately engineers the creation of a world state run by its computer’s company.^4^ This singularity theme is also abundant in SciFi movies such as *Blade Runner* (directed by Scott 1982) and *The Matrix* (directed by the Wachowskis 1999) just to mention a few.

Imaginations around replicating a brain artificially, having machines pass as humans, the possibility of them to enslave us and mergers between humans and machines indeed evoke interesting discussions around ethics, fears around technological progress as well as what it means to be a human. But what does these accounts tell us about intelligence? Apart from being possible to create artificially and revolving around simulation, passing as, or outsmarting, humans—not very much. Harari (2015, pp.152–153) avoids entering into what he labels a minefield of defining intelligence and Tegmark (2017, p. 50) only vaguely defines intelligence as the ability to accomplish complex goals (whatever complex implies here). If we look at AI success stories, they most often revolve around specific and narrow calculation tasks. In 1996 Deep Blue beat Garri Kasparow in Chess, Jeopardy was won in 2011 by IBM Watson and Lee Sedol famously lost a game of Go to the program AlphaGo in 2016. But once the use of AI is expanded to outside of the realm of narrow rule-based contexts (such as a game of chess) it becomes more problematic. If we take predictive policing systems as an example, O’Neill (2016, chapter 5) shows how the AI-powered system sends cops back to the same poor neighborhoods, creating a toxic feedback loop because policing one street creates new data that justifies more policing in that exact same street. Since every police arrest creates more data, Latinos and Blacks become more likely to be stopped in crime prevention measures in the US. At the same time, economic and predominantly white crime goes unseen for the most part. O’Neill thus concludes that predictive policing zeroes in on the poor, resulting in the criminalization of poverty and the belief that this is scientific and fair.

We are thus starting to become aware of that a narrow calculation-focused intelligence is too rigid to deal with situations in the messy realities of the socio-cultural realms humans and other organisms navigate on a daily basis. This has been picked up in *Little Britain’s* sketch *Computer Says No* in which character Carol Beer always responds to a customer's enquiry by typing it into her computer and responding with *computer says no* to even the most reasonable of requests. Teaching in a Swedish university, computers say no very often when having to use learning platforms, classroom booking systems and report grades into systems that are very rigid (to put it mildly). Another example of this rigidity is *Saga Norén,* the heroine in the popular Swedish- Danish Nordic Noir detective TV series *The Bridge.* Norén approaches everything directly and logically and is often oblivious to the fact that her blunt demeanor sometimes offends others. Norén becomes an illustration of what a world purely run by rigid AI would look like: direct and logical, but unable to empathize and account for the specificity of the situations in which she is supposed to act.

To conclude here: while important for the field of AI to kick off, not the least because of the imaginations it gives rise to, intelligence is only shallowly attended to (as also Gunkel 2020, p. 5 complains about).

AI success stories often revolve around narrow calculation-based tasks in strict rule-based contexts, but when employed in more general areas, unintended consequences arise and create problems. However, instead of avoiding the whole intelligence discussion (as Harari suggest), my proposition is to approach intelligence as different from passing and as more elaborate than solving complex problems. This leads me to the next section.

## Intelligence as “ratio” and “intellectus”

Critiquing AI is not new (see for example Dreyfus 1992; Weizenbaum 1976; Agre 1997; Hayles 1999). My contribution to this critique is by attending to fifteenth century German philosopher, theologian (appointed cardinal) and astronomer Nicholas of Cusa. While important for Catholicism, what interests me is his division of intelligence into a rational calculating part, *ratio*, and a more reflexive part, *intellectus* (Cusa 1442/2000). Being a man of God, it was important for him to acknowledge that there is a way to approach things we do not yet know about or have tangible evidence of (i.e., God). He argued that there is a possibility to scholarly approach something novel and beyond our current knowledge. He labels this approach *learned ignorance* (from his essay *De Docta Ignorancia*, see Cusa 1440/2007). To be human is to *not* know everything and being aware of this. Cusa thus suggests a conception of knowledge as infinite and learning as a never-ending process. And the more we know that we are unknowing, the more learned we become (Cusa 1440/2007, p. 9). This at the same time as we will never be able to know the full truth of everything or have all knowledge (Cusa 1440/2007, p. 11).

One is appraised to be knowing, who knows his ignorance, and only he will revere the truth, who knows that he can apprehend nothing without it (Cusa 1444/2016:16)

In this sense, Cusa believes in a reality (God) outside of human cognitive abilities and that thinking itself becomes a filter, a filter that is deeply dependent on the situation in which it is applied. *De Docta Ignorancia* is about always being open to be wrong and to acknowledge that we will never fully know.^5^

It is doubtful if not knowing can be simulated in a computer. It is thus no surprise that AI-powered automated systems do not acknowledge not-knowing, and as a consequence are incapable of dealing with the unknown. AI-powered automated systems are marked by *an imperative of total information capture* (or totalizing ambition, see Andrejevic 2020, p. 33). For an AI to make *fully accurate* predictions it needs, not only big but *all* data. However, a total system of rules, whose application to all possible eventualities is determined in advance, makes no sense. For this AI-powered automated systems either have to store and access “an infinity of facts, or having to exclude some possibly relevant facts from the computer’s range of calculations” (Dreyfus 1972, p. 170).^6^ Andrejevic (2020, pp. 115, 126) talks about this as a *fantasy of framelessness*, of a purely objective, exhaustive and definitive representation that leaves nothing out (the so-called full truth). Already Cusa (1440/2007 book 1, chs. 11–16) realized—through mathematics and geometry—that an infinity cannot be comprehended. A calculating *ratio* is dependent on well-defined categories, and these will necessarily either leave things out, or be too specific/ rigid (Cusa 1442/2000, p. 173). For example, if we count people, we will ignore differences between men, women and trans persons. But if we only count women a lot of other humans will be left out. Hence, a calculating *ratio* needs a direction and cannot account for a totality (Cusa 1442/2000, p. 174) in the same way as a *frame* is always necessarily partial (Andrejevic 2020, p. 121). Because the reality is never fixed, while categories—being based in written language—are dependent on definitions and discernments that fix them. But once something is inscribed and fixed, “all the spontaneity, mobility, improvisation, the quick responsiveness of spoken speech vanishes” (Peters 2015, p. 305). Peters gives the example of *but* and *butt* that in spoken language sounds exactly the same. It is the situation that makes us understand their different meanings. As Weizenbaum argues, “without a certain context, a frame of reference, intelligence is meaningless” (interviewed by Wendt 2015, p. 95). In written languages, there is an attempt to replace the specificity of situations with letters and signs (see also Flusser 2015). But then we end up with a mode of communication which can only deal with predefined/ pre-calculated differences and not with unfolding and unpredictable situations. Computers can only simulate processes that are described to them and transferred into some precise and rigid rules without exceptions (Dreyfus and Dreyfus 1986, pp. 53–54). Computers require precision and unambiguousness and therefore have a hard time to adapt to unfolding and unintended situations. It is this rigidity of computers that makes the Little Britain sketch (Computer Says No) so amusing.

By dividing intelligence into both ratio and intellectus, Cusa (1442/2000) underlines that calculation and rationality cannot be separated from a reflexive intellectus. To deal with the partiality, directions and framings of the categories our calculating ratio creates, we need intellectus. Intellectus relates to ratio’s categories, may examine and perhaps refine them, adapt them to the present moment, and hence make them less rigid. Our world is moving and multi-facetted, situations are constantly changing and cannot be captured or fixated once and for all. This is why we have an intellectus to handle the unknown and the uniqueness of unfolding situations. An intelligence-based solely on ratio would demand a world in which everything is known, and that is a fantasy (following Andrejevic 2020). At the same time, we cannot do without ratio as it makes our world coherent, graspable, and not in a constant confusing blurry mess.

Imaginations around AI focus on one side of the intelligence coin, ratio, and thus for the most part forget intellectus*.* Within AI, intelligence has been continuously linked to quantification—favoring logics of calculations and predictions (Engster and More 2020). This emphasis on ratio can be connected to what Kennedy (2016) describes as a *pervasive desire for numbers*. Numbers can be understood from far away and are somewhat universal as they can be shared across cultures. This desire for numbers, with its allure of objectivity and neutrality, is accompanied by an imaginary of unbiased calculation, the translation and fixation of everything into ones and zeroes. Hence AI is imagined as less biased than human intelligence and thus more reliable. Therefore, AI is used also in more socio-culturally messy areas such as policing. But the imagination that people can be predicted based on AIs making calculations on big (while not all) data, has brought with it harmful consequences. Within Critical Data Studies, researchers have shown that AIs are unfavorably biased against the poor, women, and people of color (see for example O’Neill 2016; Eubanks 2017). To be an intelligent police officer does not mean to be a replaceable cog that just *follows* a predefined manual, but to be a living apprehensive person with a reflecting intellectus that *relates to and reflects* on the manual (following Bornemark’s 2018, p. 73 reading of Cusa). Dreyfus and Dreyfus (1986) argue similarly that intelligence cannot be captured in formal rules. Instead of following predefined steps in a fixed order, intelligent humans exhibit “flexibility, judgment and intuition” (Dreyfus and Dreyfus 1986, p. 63).

This disenchantment with the ratio is not new and even has a tradition within the Social Sciences. Weber lamented on the increasing rationalization in Western societies through his metaphor of an *iron cage*. He described a disenchantment with the “belief that if we only wanted to, we could learn at any time that there are, in principle, no mysterious unpredictable forces in play, but that *all things—in principle—can be controlled through calculation*” (1922/2008, p. 35, emphasis in original). The iron cage thus traps individuals in systems based purely on teleological efficiency, rational calculation and control. You could argue that AI with its one-sided focus on rationality and calculation constitutes such an iron cage.^7^

## The need for an organic body

As Cusa underlines the role of intellectus when facing situations as they unfold in the present moment, it could be argued that he is forwarding an *organic* view of intelligence. According to Cambridge Dictionary (2020) organic implies developing naturally over time *without being forced or planned*, as well as *derived from living matter*. As AI is imagined today it is most often as disembodied, or at least lacking an *organic* body. This has to do with locating intelligence inside the brain and imagining it possible to engineer a brain artificially as discussed previously. Artificial is seen as opposite to natural (living matter) and thus also in opposition to organic. But as Gunkel (2020, p. 8) underlines, this does not mean that artificial equals fake, but rather that artificial implies simulation of an organic phenomenon. In this sense, it is the simulation connotation to artificial that is in opposition to organic, as something organic cannot be planned or forced.

The importance of an organic body for intelligence has been highlighted for some time (Dreyfus 1972; Rosch et al. 1991; Damasio 1994/2005; Penny 2013) and reducing intelligence to formal manipulation of symbols in the quest of simulating humans and human behavior have also been thoroughly criticized (Hayles 1999). Still, popular accounts of AI today are full of imaginations that it would be possible to get rid of our organic body. As Hayles (1999, p. 1) argues, the belief that information can circulate unchanged among different material substrates^8^ is a defining characteristic of the cultural moment we find ourselves in. In Harari’s (2015) account of *Homo sapiens* evolving into *Homo deus*, we would be able to escape old age and death which are both blamed on our organic decaying bodies. In this sense disembodiment implies immortality through simulation. Tegmark’s (2017) Life 3.0. is also an imagination in which the body becomes irrelevant. Life 1.0 is about survival and replication. Life 2.0 has the ability to design its own software, knowing how to process information and acting upon it. Life 3.0 will have the ability to even design its own hardware, i.e., the body, and voilà we will be free from evolutionary shackles. Tegmark (2017, p. 55) even claims that the conventional wisdom among AI researchers is that intelligence is about information and computation, not about the flesh. This is about privileging informational patterns over material instantiation so that embodiment is seen as an accident of evolution rather than an inevitability of life (Hayles 1999, p. 2). The body’s materiality becomes secondary to the logical semiotic structures it encodes (Hayles 1999, p. 192). It is when information loses its body, that it gives way to what Hayles (1999) labels a *posthuman*, a pivotal move according to her for equating humans with computers.

It is possible to draw a line from these imaginations to tech culture’s origin in the 60’s counter-cultural movement and hippie influence, via the hacker. The out-of-body experience, induced by foremost LSD and other psychedelic drugs, greatly impacted how some computer pioneers imagined the future and the role of computers in it (Turner 2006). It was believed that LSD allowed them to escape their bodies and experience a kind of shared consciousness, the same with computer-mediated communication. To enter *cyberspace,* you needed to forsake your own body and become information (Turner 2006). This was to be picked up in *The Declaration of the Independence of Cyberspace* in which it is stated that “there is no matter here” and that “our identities have no bodies” (Wikipedia 2020). Indeed, cyberspace is generally considered a space without organic bodies.

SciFi has been tremendously important for such imaginations. Disembodiment is a central theme in *Neuromancer* (Gibson 1984). Protagonist Henry Dorsett Case jacks himself “into a custom cyberspace deck that projected his *disembodied* consciousness into the consensual hallucination that was the matrix” or how Case “lived for the *bodiless* exultation of cyberspace” or how he “fell into the *prison of his own flesh*” for him the worst kind of punishment (Gibson 1984, p. 6, emphasis added). Just like Harari’s *Homo deus*, Neuromancer is all about mixing biology with computing, upgrading the human with data, or downgrading it with organic flesh.

However, intelligence seems to develop organically and be linked to organic bodies. One interesting example is how research on plants have revealed that they can make rational decisions, learn (make associations) and even have a memory. This has led evolutionary ecologist Gagliano (2018, p. 67) to the conclusion that we have an obsession with the brain when discussing intelligence. She has shown that a plant that has no brain, such as the *Mimosa Pudica*, can be taught that a disturbance (such as a drop from a height) is unharmful. After a few drops, the plants learned not to close their leaves and also remembered this several days after the initial drops (Gagliano 2018, p. 59). Gagliano (2018, p. 64) thus proved that mimosas have the faculty of memory, and their behavior is not hard-wired in their DNA (as originally thought) since they can learn from experience. What this line of research suggests is that intelligent action is not exclusive to organisms with a brain, but rather depending on handling situations, organically, as they unfold in the present moment.

Within neurology, researcher, and best-selling author Damasio has underlined the importance of the *body-proper* (i.e., the body excluding the brain) for rationality, consciousness, and the mind. The function of the brain/mind is to be informed about what goes on in the body-proper and the surrounding environment. Damasio (2003/2004, p. 190) thus connects brain/mind and body-proper together, conceiving of the mind as arising from (or in) a brain that is connected to a body-proper with which it interacts. He argues that the mind is closely shaped by the body and also destined to serve it. “No body, never mind” (Damasio 1999, p. 143) as he eloquently puts it. He, therefore, suggests turning the famous cartesian principle around. Instead of *I think, therefore I am* he suggests *I am* (i.e. have a body), *therefore I can think* (Damasio 1994/2005, p. 248). Such reasoning should put an end to the disembodied AB (Artificial Brain) projects out there. Indeed, research has shown that data-processing takes place all over the organic body, thus undermining Cartesian/cognitivist assertions that thinking only occurs in a brain (Penny 2013, p. 154).

Within tech philosophy the argument for a body handling situations as they unfold in the present moment has also been made. Dreyfus (1972) was early to critique the imagination that intelligence would solely depend on what he called symbolic manipulation. This is the belief that the brain processes information by way of some biological equivalent of on/off switches (what he labels the *biological assumption*) and that the mind can be viewed as a computing device operating on bits of information according to formal rules (the *psychological assumption*, Dreyfus 1972, p. 68). Instead, Dreyfus underlines the role of the body “in organizing and unifying our experience” (Dreyfus 1972, p. 146). What distinguishes humans from machines “is not a detached, universal, immaterial soul, but an involved, self-moving, material body” (Dreyfus 1972, p. 148). To be intelligent we need to be *in* this world (according to Fjelland’s 2020 reading of Dreyfus). To understand a person is not to look into that person’s brain or mind, but to walk in her shoes. Our bodies organically handling situations as they unfold in the present moment allow us to dwell in the world while at the same time avoid formalizing everything. The body helps us modifying our expectations in its ongoing evaluation and reevaluation of the situations we find ourselves in (Dreyfus 1972, p. 162). Dreyfus and Dreyfus (1986, preface xx) talk about this as *know-how* (in contrast to *know-what*) underlining what they label as *intuitive intelligence*. Fjelland (2020), in his updated reading of Dreyfus argument, connects this to *tacit knowledge* and Aristotle’s concept of *prudence*, the ability to make right decisions in concrete situations. Such intuitive intelligence (prudence or tacit knowledge) allows us to skillfully navigate our everyday changing environment and unfolding situations. In a given situation, not everything is relevant to consider as already Dreyfus (1972, p. 170) argued, underlining the situational character of relevance.

Dreyfus highlighting of intuitive intelligence directs me to the importance of the *nonconscious* as we deal with unfolding and unknown situations in the present moment. The importance of *nonconscious cognition* is something Hayles (2017) discusses in her more recent work. Her aim is to tease out a definition of cognition that applies to both technical systems and biological life forms (while excluding material processes) and to balance what she sees as an excessive focus on consciousness/awareness. I think we can all agree that AI-powered automated systems are nonconscious/unaware. My argument is that part of nonconscious cognition, what Dreyfus describes as intuitive intelligence, needs an organic body to develop. Hence, it is not possible to equate what Hayles labels as nonconscious cognition with intuitive intelligence because of her attribution of nonconscious cognition to *both* technical systems and organic cognitizers. She acknowledges that there are differences between the two but merely underlines that they perform similar functions (Hayles 2017, p. 13). Her argument for the importance of nonconscious cognition thus needs to be refined.

Presence is an important concept in my argument for organic intelligence, handling the unknown situations as they unfold in the *present* moment. In her earlier works, Hayles (1999, p. 29) argued that simulation and dematerialization are informed by a shift toward pattern (vs randomness) away from *presence* (vs absence). It would be interesting to hear her reason about the neglected presence in technical systems and whether this does not impact the development and deployment of nonconscious cognition. When displacing presence by pattern (see Hayles 1999, p. 40), I would argue that intelligence is lost. To act intelligently we need to be organically in the present moment to handle its uniqueness, its unknown aspects. The question that Hayles leaves unanswered is how the cognitive unconscious of technical systems has developed given their preference for patterns and lack of organic bodies.

To be fair, Hayles differentiates between intelligence and cognition. Cognition is about processing information and acting upon such processing, while intelligence is about having a goal and pursuing it (Hayles 2017, pp. 51–52). Following this, AI would be a cognitizer, but not an intelligent one. At the same time, a focus on intelligence as goal-oriented tilts towards the ratio-side in Cusa’s two-sided intelligence conception. Cusa’s emphasis on not-knowing is about how to approach new or unfolding situations, while nonconscious cognition is about what we already know, but what has been internalized, pushed to the background so that the consciousness will not be overwhelmed (Hayles 2017, p. 10).

## Conclusion

To act intelligently we need to handle the contexts and situations in which we find ourselves. But these situations are always changing and cannot always be anticipated. And what cannot be anticipated cannot be accounted for by AI-powered automated systems given its fantasy of *framelessness* and imperative of total information capture (Andrejevic 2020). The key to this critique of AI is the importance of not-knowing, which is different from Hayles (2017) cognitive nonconscious. In other words, intelligent action is not only teleologically/goal-oriented but situational, embedded in the handling of the contingencies of the present and unfolding moments. The question then becomes whether AI is an oxymoron. Will computers ever learn to handle the messiness of the present? Will inorganic machines be able to conquer intellectus, and if so, can this be done through data? I am skeptical. Intelligence is a complicated matter, has no single dimension, and seems to include a time-bound organic body acting in a present moment and in a changing environment.

Proposing that AI is an oxymoron is an intertextual reference to Gitelman’s anthology from 2013 *raw data is an oxymoron*, which in turn is a quote from Bowker (2005). Bowker (2013, p. 168) was in his turn referencing Lévi-Strauss in which the natural is seen as *raw*, and the social as *cooked*. However, data is not natural, most often it is *captured*, extracted through observations and computation. Behind data production, there are sophisticated assemblages of people, places, documents, practices and technologies, making data a product of complex processes to be useful for the contexts in which it appears. Indeed, data are both social (situated in a context) and material in that it has a form. In terms of computer data, this would be in the form of bits stored on a hard drive and depending on infrastructures such as data centres and broadband cables (Holt and Vonderau 2015). The conclusion is that data is never raw, should be ‘cooked with care’ (Bowker 2005, p. 184), otherwise it might ‘rot’ (Boellstorff 2013) and thus in need of ‘repair’ (Pink et al. 2018). As raw is supposed to be in an oxymoronic relationship to contextual and situated data, it seems artificial is in an oxymoronic relationship to genuine, authentic, present and organic intelligence.

As stated in the introduction, imaginaries are productive and cannot be dismissed as a mere fetish or false believes (Bucher 2017, p. 31). It is therefore important not to leave it to mathematicians, computer scientists and software engineers to define intelligence for us. Because if we do, we risk unempathetic rigid AI-powered automated systems employed in even more areas of the messy socio-cultural realms we navigate on a daily basis. Let computers focus on ratio-stuff and organic cognitizers reign in the realms that demands an intellectus. Following Dreyfus and Dreyfus (1986, p. 205) I suggest we should reject the imagination of humans as information processing devices and realize the limits of AI and its overreliance on the computer metaphor. At the same time, an overtly anthropocentric view of intelligence is also unattractive. We live in a world and in a time in which the limits of human ingenuity are painfully apparent with climate change and increasing intolerance as prime examples.

As I am writing this quarantined in my home office due to Covid-19, the importance of an organic body acting in a present moment together with other human bodies has become acutely apparent. This brings me back to Wyclef Jean and the opening scene of this article at SXSW2019. There is indeed nothing that can beat an organic body interacting with, and reacting to, other organic bodies in an unfolding situation and changing environment. As my recent course evaluations bare witness off, there is nothing that beats a professor teaching live in a lecture hall to students which reacts and interacts with the professor, no Zoom, no Microsoft Teams, no nothing.

## Data Availability

Not applicable.

## Footnotes

1 Dreyfus (1972, preface xv) goes even further back and locates the idea of reducing all reasoning to calculation to when the Greeks invented logic and geometry.

2 you may wonder if his obsession with imitation and passing had to do with him being gay in the UK during the second world war.

3 an interesting objection here is Bostrom (2014, pp. 54–56) who points at the risks of medical complications of what he labels *brain-computer interfaces*, or *superintelligence through cyborgization*, and also underlines the benefits of keeping machines outside of our bodies (for updating and maintenance purposes).

4 Interesting that Tegmark’s AI is labelled Prometheus, when Prometheus in the Greek myth was a friend of humans, went against the will of the Gods in order to help humans. In Tegmarks tale this would have implied that his AI would rebel against the computer company and its imaginary Omega team.

5 Here it is possible that Cusa was inspired by Socrates who allegedly should have said that *I know that I am intelligent, because I know that I know nothing* and *the only true wisdom is in knowing that you know nothing.*

6 furthermore, these “facts” are never context free as Dreyfus and Dreyfus (1986, p. 65) argued, and later picked up by Gitelman (2013) and others in her anthology *Raw Data is an Oxymoron.*

7 see also Dreyfus and Dreyfus (1986, pp. 195–196) lamenting the increasingly bureaucratic nature of society and its overreliance of calculative rationality, sacrificing know-how, wisdom and judgment in the process.

8 Ribes and Jackson (2013) illustrates nicely how this is not the case concerning data production as behind it there are complex assemblages of people, places, documents, practices and technologies, making data a product of intricate processes in order to be useful for the contexts in which it appears.
